# Case study: Effect of surgical metal implant on single frequency bioelectrical impedance measures of an athlete

**DOI:** 10.14814/phy2.14464

**Published:** 2020-05-30

**Authors:** Dale R. Wagner

**Affiliations:** ^1^ Kinesiology & Health Science Department Utah State University Logan UT USA

**Keywords:** bioimpedance, body composition, fat, fat‐free mass, prosthesis

## Abstract

This case study examined the influence of a surgical metal implant on the bioelectrical impedance analysis (BIA) readings of an athlete. Single‐frequency BIA using a tetrapolar electrode configuration was applied to both the right and left sides of a 23‐year‐old female jumper who had an 8 × 345 mm titanium alloy nail implanted in her left tibia. The metal implant reduced BIA resistance and reactance on the implanted side by 27 and 6 ohms, respectively. This reduction in impedance resulted in a 0.4 kg–1.9 kg increase in the estimate of fat‐free mass (FFM) depending on the prediction formula used. There was a concomitant decrease in the estimate of body fat percentage (%BF) with the underestimation ranging from 0.6% to 2.7% BF depending on the prediction formula. A metal implant of substantial size can alter the BIA reading. Technicians should apply BIA to the opposite side of the body when athletes present with a surgical implant in a limb.

## Graphical abstract

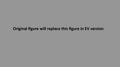

## 1 INTRODUCTION

A healthy body composition, or an appropriate amount of fat mass (FM) relative to total body mass (BM), is important for general well‐being as well as athletic performance. Body composition is of particular concern, and vital to success, in gravitational sports in which one must move against gravity, sports with weight classes, and aesthetic sports in which the athlete's body shape may influence the scoring (Ackland et al., 2012).

One of the most commonly used methods to assess body composition is bioelectrical impedance analysis (BIA). Single frequency BIA sends a weak electrical current through the body and measures the impedance to that current flow. The resistance or impedance value, combined with other variables such as height, are used to estimate fat‐free mass (FFM). The principles, as well as the strengths and limitations, of the BIA method specific to measuring the body composition of athletes have been reviewed (Kerr & Hume, 2018; Moon, 2013).

BIA is a popular body composition method because the device is portable, and the procedure is fast, painless, and easy to administer (Kerr & Hume, 2018). Unfortunately, the validity of this method can be compromised by recent exercise and acute changes in hydration status (Kerr & Hume, 2018). Despite a wealth of documentation on factors that can alter BIA readings, there is sparse information available regarding the influence of metal implants; thus, the purpose of this case study report.

## 2 CASE REPORT

This was a case study of a 23‐year‐old female jumper competing on a Division I National Collegiate Athletics Association (NCAA) track and field team. Four and a half years prior to this data collection, the subject had an 8 x 345 mm Stryker T2 nail surgically implanted in her left tibia as treatment for a chronic anterior tibial stress fracture. The Stryker T2 nail is a titanium alloy that contains aluminum, vanadium, and iron (personal communication, Stryker, Inc.).

The subject was informed of the intent to use her data for a published case study. The university's Institutional Review Board approved the data collection, and the subject signed a written informed consent as well as an authorization for the use of protected health records.

Height was measured to the nearest 0.1 cm with a wall‐mounted stadiometer (Seca 216, Seca Corp.), and weight was measured to the nearest 0.1 kg with a digital scale (Seca 869, Seca Corp.). The subject was wearing only a t‐shirt and shorts for the data collection.

The subject laid supine on a nonconducting treatment table with arms and legs abducted to approximately 30° to 45° from the trunk for 5 min. This time frame is adequate for the total body water to stabilize (Gibson, Beam, Alencar, Zuhl, & Mermier, 2015). During this time, the dorsal surface of the wrists, hands, ankles, and feet were cleaned with an alcohol wipe. Electrodes were applied in a tetrapolar configuration on both sides of the body using anatomical landmarks suggested by the manufacturer: (a) the superior borders of detecting electrodes of the wrist and ankle at the level of the ulnar head and medial malleolus, respectively, and (b) signal electrodes at the metacarpal‐phalangeal joint of the middle finger and at the base of the metatarsal‐phalangeal joint of the second toe.

A single‐frequency BIA machine operating at 50 kHz (Quantum II, RJL Systems, Clinton Township) was used to apply the current and read the resistance. Measurements were made in duplicate on both sides of the body. The multiple readings were done in quick succession, and the subject remained in the same position throughout.

In addition to the resistance and reactance values obtained directly from the analyzer, FFM was estimated using the equations of Fornetti, Pivarnik, Foley, and Fiechtner (1999), Lohman (1992), and Sun et al. (2003) (Table 1). The Fornetti et al. (1999) formula was chosen because it is specific to female collegiate athletes. In a review of BIA for athletes, Moon (2013) recommended the BIA formula of Lohman (1992) specific to active females aged 18–35 years. Finally, the Sun et al. (2013) formula was selected because it is a commonly used, general‐population BIA formula that has been cited over 400 times and was validated against a multicomponent model. Subsequent to calculating FFM, FM was determined by BM–FFM, and body fat percentage (%BF) was calculated as (FM/BM) × 100.

**TABLE 1:** Bioelectrical impedance (BIA) formulas used to estimate fat‐free mass (FFM)

Citation	Formula
Fornetti et al. (1999)	FFM (kg) = 0.282 (ht) + 0.415 (wt) – 0.037 (*R*) + 0.096 (*Xc*) – 9.734
Lohman (1992)	FFM (kg) = 0.666 (ht^2^/*R*) + 0.164 (wt) + 0.217 (*Xc*) – 8.78
Sun et al. (2003)	FFM (kg) = −9.53 + 0.69 (ht^2^/*R*) + 0.17 (wt) + 0.02 (*R*)

Abbreviations: FFM, fat‐free mass in kg; ht, height in cm; *R*, resistance in ohms; wt, weight in kg; *Xc*, reactance in ohms.

The subject had a height and weight of 173.3 cm and 70.3 kg, respectively. Although, repeated measures on the same side of the body were consistent to within ± 1 ohm, the resistance and reactance from the subject's left side (tibia containing the metal implant) were substantially less than the right side (Table 2). Consequently, this led to a larger estimate of FFM when BIA was applied to the implant side, and a reduction in the %BF estimation of 0.6%BF to 2.7%BF depending on the prediction equation used.

**TABLE 2:** Single‐frequency tetrapolar BIA comparison of left and right sides with a surgical metal implant on the left side

	Left side (metal implant)	Right side (no metal)	Difference (left minus right)
Resistance (ohms)	469	496	−27
Reactance (ohms)	59	65	−6
FFM_Fornetti_ (kg)	56.6	56.2	+0.4
FFM_Lohman_ (kg)	58.2	57.2	+1.0
FFM_Sun_ (kg)	56.0	54.1	+1.9
%BF_Fornetti_ (%)	19.5	20.1	−0.6
%BF_Lohman_ (%)	17.2	18.6	−1.4
%BF_Sun_ (%)	20.3	23.0	−2.7

Abbreviations: %BF, body fat percentage; FFM, fat‐free mass.

## 3 DISCUSSION

A metal implant running the length of the subject's shank reduced the BIA resistance and reactance compared to the nonaffected side. This is logical because of the conductive properties of metal. The extent to which this reduction in electrical impedance had on estimates of FFM and %BF varied depending on the prediction equation. Interestingly, the prediction formulas of Fornetti et al. (1999) and Lohman (1992), which both include reactance as an independent variable, resulted in the least amount of variability between right and left side measurements. The metal implant reduced reactance by 9.2%, while resistance declined by only 5.4%. More research is needed to determine if metal implants consistently have a proportionally greater influence on reactance than resistance or if this was unique to this case study. Reactance, which is often absent in many generalized BIA prediction formulas, might be an important variable in limiting the error introduced when a metal implant is present.

It is important to note that the amount of metal embedded in the subject was substantial. The length and mass, anatomical location, and metal composition (e.g., titanium, steel, etc.) of a surgical implant are likely variables that could influence the extent to which a metal rod or pin effects the resistance and reactance to electrical current. For example, a metal plate or screws at the clavicle might have negligible influence on BIA results, especially if the implant is not in the direct path of the electrical current.

This is the first known report on the influence of a metal implant on the BIA results of an athlete. However, Steihaug and colleagues (2017) applied single frequency, tetrapolar BIA to both the fractured and unfractured sides of hip fracture patients aged 80 ± 8 years. In the immediate postoperative period, there was a significant difference (*p* < .001) in resistance between the fractured hip with the new implant (496 ± 98 ohms) versus the unfractured hip (527 ± 101 ohms). However, at the 3‐month follow‐up, the mean difference in BIA resistance between sides was only 3 ohms and not significant (*p* = .40), suggesting that the trauma (swelling) from surgery had more influence on the BIA results than the implant. Additionally, they commented that the BIA reading did not differ across the type of implant (cannulated screws, compression screw, or hip arthroplasty). Given the contradictory findings from Steihaug, Bogen, Kristoffersen, and Ranhoff (2017) with the present case study, more research is warranted with a range of subject ages, implants of various types and sizes in different anatomical locations, and types of BIA analyzers (e.g., single frequency, multifrequency, bioimpedance spectroscopy) or electrode configurations (e.g., tetrapolar, 8‐segment).

### 3.1 Practical application

It is atypical for a young athlete to have a metal implant, and thus the reason for this case report. However, given modern surgical techniques, this scenario might become more common. The BIA technician should get into the habit of asking if an athlete has a surgical implant and if so, the date of surgery to rule out postsurgery edema effects. Even if a metal implant might have small to negligible effect, it is prudent to administer the BIA test with a tetrapolar configuration on the contralateral side. Additionally, consistency in applying BIA to the same side of the body is important when tracking changes.

## 4 CONCLUSION

A titanium alloy surgical nail running through the shaft of the tibia of a female track and field athlete reduced BIA resistance and reactance by 5.4% and 9.2%, respectively. This resulted in a marginal to small increase in the estimation of FFM and decrease in %BF for the implanted side relative to the nonsurgical side. BIA technicians should be cognizant of the potential impact that metal surgical implants can have on BIA results.

## CONFLICT OF INTEREST

This research was conducted without external funds, and the author declares no conflict of interest.

## References

[phy214464-bib-0001] Ackland, T. R. , Lohman, T. G. , Sundgot‐Borgen, J. , Maughan, R. J. , Meyer, N. L. , Stewart, A. D. , & Müller, W. (2012). Current status of body composition assessment in sport. Sports Medicine, 42, 227–249. 10.2165/11597140-000000000-00000 22303996

[phy214464-bib-0002] Fornetti, W. C. , Pivarnik, J. M. , Foley, J. M. , & Fiechtner, J. J. (1999). Reliability and validity of body composition measures in female athletes. Journal of Applied Physiology, 87, 1114–1122. 10.1152/jappl.1999.87.3.1114 10484585

[phy214464-bib-0003] Gibson, A. L. , Beam, J. R. , Alencar, M. K. , Zuhl, M. N. , & Mermier, C. M. (2015). Time course of supine and standing shifts in total body, intracellular and extracellular water for a sample of healthy adults. European Journal of Clinical Nutrition, 69, 14–19. 10.1038/ejcn.2013.269 24398638

[phy214464-bib-0004] Kerr, A. , & Hume, P. A. (2018). Non‐imaging method: Bioelectrical impedance analysis In Hume P. A., Kerr D. A., & Ackland T. R. (Eds.), Best practice protocols for physique assessment in sport (pp. 101–116). Singapore: Springer.

[phy214464-bib-0005] Lohman, T. G. (1992). Advances in body composition assessment. Champaign, IL: Human Kinetics.

[phy214464-bib-0006] Moon, J. R. (2013). Body composition in athletes and sports nutrition: An examination of the bioimpedance analysis technique. European Journal of Clinical Nutrition, 67, S54–S59. 10.1038/ejcn.2012.165 23299872

[phy214464-bib-0007] Steihaug, O. M. , Bogen, B. , Kristoffersen, M. H. , & Ranhoff, A. H. (2017). Bones, blood and steel: How bioelectrical impedance analysis is affected by hip fracture and surgical implants. Journal of Electrical Bioimpedance, 8, 54–59. 10.5617/jeb.4104

[phy214464-bib-0008] Sun, S. S. , Chumlea, W. C. , Heymsfield, S. B. , Lukaski, H. C. , Schoeller, D. , Friedl, K. , … Hubbard, V. S. (2003). Development of bioelectrical impedance analysis prediction equations for body composition with the use of a multicomponent model for use in epidemiologic surveys. American Journal of Clinical Nutrition, 77, 331–340. 10.1093/ajcn/77.2.331 12540391

